# Pushing the boundaries: *Science Advances*

**DOI:** 10.1126/sciadv.1701534

**Published:** 2017-06-07

**Authors:** Jeremy Berg

**Affiliations:** Editor-in-Chief, Science Family of Journals

**Keywords:** editorial, open-access, journal publishing

**Figure:**
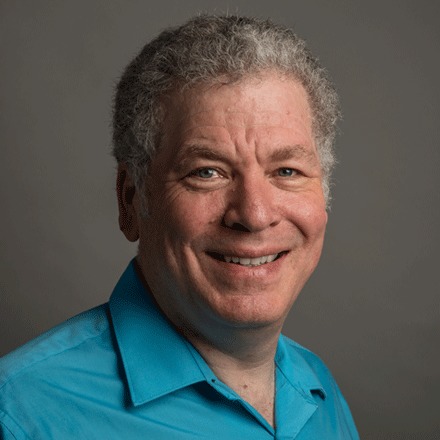
Jeremy Berg

S*cience Advances* was launched on February 2015 at the American Association for the Advancement of Science (AAAS) annual meeting in San Jose, California, as the online, open-access expansion of *Science* magazine. *Science Advances*, like its older sibling, covers the full gamut of scientific disciplines, including (but not limited to) earth and space sciences; ecology, evolution, and environmental biology; biomedical, biological, and neuroscience; social sciences; and chemical, computational, mathematical, and physical sciences as well as applied sciences and engineering. We publish research articles and reviews that illuminate the leading edge of national and international research, both within and across scientific disciplines.

The foundation of *Science Advances* is an outstanding editorial team of active scientists. With expertise spanning the many fields that we cover, the journal’s editorial resources are anchored in communities of practicing researchers. This community base places each Board member in the midst of a vibrant area of innovative research, providing them with informed views of the territories where exciting new knowledge may emerge. Our editorial team has almost doubled since our launch in 2015, growth needed to extend the depth and breadth of the science reflected in the dramatically increasing volume of our submissions. Our exemplary editorial team is committed to identifying, recruiting, and publishing research that significantly advances the boundaries of knowledge in meaningful and substantive ways, science that can have substantial impact at broad and at discipline-specific scales, both within and across disciplines.

In addition to original research articles, *Science Advances* also publishes invited reviews. In these formats, we ask experts to go beyond cataloging progress in a particular field and, instead, to synthesize existing knowledge and highlight concurrent—and sometimes opposing—views of current trends or evolving directions. We also publish special series in areas of rapid development, such as “Materials by Design” and “Advances in Bioelectronics.” We are actively developing additional topics for these kinds of focused collections. In keeping with the mission of AAAS, our publisher, we value rigorous studies that have implications for public policy that can, in turn, advance science, engineering, and innovation throughout the world.

The breadth and depth of scientific areas we cover are made possible in part by the opportunities afforded by digital, open-access publishing. *Science Advances* has flexible formats and often publishes longer papers with greater depth than those typically found in *Science*, appropriate for cutting-edge research that demands the presentation of a broader scope of experiments or of a finer level of detail. By virtue of our digital format, *Science Advances* is relatively unconstrained in the topical balance we cover from one issue to the next: The mix of disciplines we publish twice a week is somewhat organic, largely reflecting the breadth and quality of manuscripts submitted and accepted.

Our publications since launch reflect both needs for high-quality publishing venues in certain areas and our appetite for papers across all of the disciplines we cover, including cross-, inter-, and trans-disciplinary work; therefore, the distribution of what we publish in various domains may be higher or lower in any one issue, or quarter, or year. We are still growing substantially, as more researchers read and cite our published papers and come to know *Science Advances* as the high-quality open-access journal that it is.

*Science Advances* is attracting an enormous volume of submissions, many more excellent manuscripts than we can publish. Since our launch, we have received over 7000 submissions from over 60 countries and have published nearly 1250 research articles and reviews. All of these papers reflect our flexibility, broad scope, and our high selectivity, all anchored in our commitment to publish impactful, reproducible science. We are delighted and excited by the tremendous growth of *Science Advances* and look forward to continuing to pursue our goal of pushing the boundaries of knowledge forward in the years to come.

